# Colitis-associated colorectal carcinogenesis: molecular mechanisms and therapeutic potential

**DOI:** 10.1186/s43556-026-00623-0

**Published:** 2026-10-15

**Authors:** Yi Lu, Xixi Wang, Cuike Yin, Jiaxuan Wang, Shaoqing Guo, Zihan Wang, Jing Zhou, Mengyuan Cui, Xiaoxia Cheng, Lei Zhang, Changjiang Qin

**Affiliations:** 1 Department of General Surgery, The Second Affiliated Hospital of Henan University (Huaihe Hospital), Henan University, Kaifeng, Henan 475000 China; 2 School of Basic Medical Sciences, Henan University, Kaifeng, Henan 475004 China

**Keywords:** Colitis-associated colorectal cancer, Chemokines, Gut microbiota, Tumor microenvironment, Immune dysregulation, Targeted therapy

## Abstract

Chronic colitis is a major risk factor for colorectal cancer (CRC), particularly for colitis-associated colorectal cancer (CAC), where persistent inflammation drives the transition from mucosal injury to malignant transformation. Accumulating evidence indicates that gut microbiota dysbiosis, epithelial barrier disruption, microbial translocation, chronic immune activation, and aberrant inflammatory signaling collectively contribute to CAC initiation and progression. Sustained inflammation promotes oxidative stress, DNA damage, genetic/epigenetic alterations, and tumor microenvironment remodeling, ultimately facilitating tumor development and immune evasion. These processes form an interconnected pathogenic network linking microbial, inflammatory, genetic, immune, and stromal alterations. This review comprehensively summarizes the key drivers and molecular mechanisms of CAC, covering microbiota and barrier dysfunction, inflammatory signaling, oxidative stress and DNA damage, genetic and epigenetic alterations, and tumor microenvironment remodeling and immune dysregulation. We further review recent advances in diagnostic biomarkers, disease monitoring, chemoprevention, targeted therapies, and emerging interventions, including the potential utility of specific chemokine axes as biomarkers and therapeutic targets. Particular emphasis is placed on the translational challenges of linking mechanistic insights to diagnostic and therapeutic advances, including biomarker validation, stage-specific interventions, and patient stratification across disease progression. By integrating these interconnected mechanisms and therapeutic strategies, this review provides a comprehensive framework for understanding CAC pathogenesis and identifies opportunities for precision prevention, early detection, and multimodal treatment.

## Introduction

Colorectal cancer (CRC) is the third most common malignancy and the second leading cause of cancer-related mortality worldwide [1]. Among the diverse etiological subtypes of CRC, colitis-associated colorectal cancer (CAC) is a distinct inflammation-driven malignancy arising from chronic intestinal inflammation, particularly in inflammatory bowel disease (IBD) patients, including ulcerative colitis (UC) and Crohn’s disease (CD) [2, 3]. Epidemiological studies have shown that patients with UC or CD have approximately a two- to threefold increased risk of developing CRC [4, 5]. CAC is a long-term complication of IBD, and its risk increases with the duration and extent of intestinal inflammation. CAC is characterized by distinct clinicopathological features, including multifocal dysplasia and flat or inconspicuous lesions [6, 7]. Although CAC and sporadic CRC share similar histopathological features, they arise via distinct biological processes [3, 8]. Unlike sporadic CRC, which follows the conventional adenoma–carcinoma sequence, CAC develops within persistent intestinal inflammation via an inflammation–dysplasia–carcinoma pathway [9]. Chronic inflammation perpetuates aberrant immune activation, drives genetic and epigenetic alterations, and elicits molecular features that distinguish CAC from sporadic CRC [10, 11]. These unique features underscore the necessity of understanding CAC as a distinct inflammation-driven subtype and provide the rationale for exploring its underlying mechanisms and therapeutic vulnerabilities.

CAC development is orchestrated by interconnected inflammatory, microbial, immune, and stromal processes that collectively drive malignant transformation [12, 13]. Microbial translocation and chronic activation of innate and adaptive immune responses establish a pro-inflammatory microenvironment that promotes oxidative stress, DNA damage, epigenetic alterations, and aberrant activation of oncogenic pathways, thereby accelerating malignant transformation [14–16]. These interconnected processes also reshape the TME and immune landscape, contributing to sustained epithelial injury, abnormal proliferation, and immune evasion. Chemokines and their receptors function as important regulators within this network by coordinating immune cell recruitment, amplifying inflammatory responses, and contributing to TME remodeling [17, 18]. Dysregulated chemokine networks not only mediate infiltration of immunosuppressive cells, including tumor-associated macrophages (TAMs), myeloid-derived suppressor cells (MDSCs), and regulatory T cells (Tregs), but also contribute to epithelial–mesenchymal transition, immune evasion, angiogenesis, and metastasis [19–21]. Increasing evidence has also identified chemokines, inflammatory mediators, microbial signatures, and immune-related molecules as potential biomarkers and therapeutic targets in CAC [15, 22].

To provide an integrated view of inflammation-associated colorectal carcinogenesis, this review first discusses the key drivers of CAC initiation, including gut microbiota dysbiosis, epithelial barrier disruption, microbiota-associated inflammation, and chemokine-mediated immune cell recruitment. It then summarizes the major molecular mechanisms underlying malignant transformation, focusing on inflammatory signaling pathways, oxidative stress and DNA damage, genetic and epigenetic alterations, and tumor microenvironment remodeling and immune dysregulation. The review next examines current diagnostic and monitoring strategies, followed by recent advances in chemoprevention, targeted therapies, and emerging interventions. Collectively, this framework aims to clarify the interconnected mechanisms linking chronic colitis to colorectal carcinogenesis and highlight potential opportunities for the prevention and treatment of CAC.

## Key drivers of inflammation-associated colorectal carcinogenesis

### Inflammatory responses driven by gut microbiota dysbiosis and epithelial barrier disruption

#### Gut microbiota dysbiosis and its tumor-promoting effects

The gut microbiota plays a central role in maintaining intestinal homeostasis by regulating metabolic balance, immune equilibrium, and epithelial barrier integrity. Alterations in its composition and function have been recognized as major driving factors in the initiation and progression of CAC [23]. In patients with IBD and CRC, the gut microbiota commonly exhibits reduced microbial diversity, depletion of beneficial commensal bacteria, and enrichment of opportunistic pathogens [24–26]. Among these alterations, expansion of Proteobacteria and reduction of Firmicutes are considered characteristic features of inflammation-associated dysbiosis, while certain low-abundance commensals may acquire pathogenic properties under chronic inflammatory conditions, thereby perpetuating intestinal inflammation [24, 27].

Several specific microbial species have been directly implicated in colorectal tumorigenesis. Fusobacterium nucleatum is significantly enriched in CRC tissues and promotes tumor cell proliferation and persistent inflammation through activation of the Wnt/β-catenin and NF-κB signaling pathways via its FadA adhesin, as well as through immune evasion mediated by the Fap2 protein [28, 29]. Enterotoxigenic (ETBF) secretes the BFT toxin, which cleaves E-cadherin and activates β-catenin signaling, thereby inducing abnormal epithelial proliferation and inflammatory cytokine production [26, 30]. In addition, strains harboring the pks genomic island produce colibactin that induces DNA double-strand breaks, whereas Enterococcus faecalis promotes genomic instability and tumor transformation through oxidative stress and pro-inflammatory signaling [29, 30].

Beyond bacterial pathogens themselves, microbiota-derived metabolites also play critical roles in regulating tumorigenesis. Secondary bile acids, including deoxycholic acid (DCA) and taurodeoxycholic acid, promote tumor development by activating NF-κB and Wnt/β-catenin signaling pathways and inducing oxidative DNA damage, whereas short-chain fatty acids (SCFAs), particularly butyrate, exert anti-tumor effects through histone deacetylase (HDAC) inhibition, induction of apoptosis, and maintenance of epithelial homeostasis [29, 31]. Furthermore, tryptophan metabolites contribute to intestinal immune homeostasis through activation of the aryl hydrocarbon receptor (AhR), and reduced levels of these metabolites are closely associated with persistent inflammation and epithelial barrier dysfunction [24, 28]. Dysbiosis-associated metabolic reprogramming may further disrupt immune balance and promote the establishment of a pro-tumorigenic microenvironment [32].

#### Epithelial barrier disruption and microbial translocation

Gut microbiota dysbiosis not only directly induces pro-inflammatory and pro-tumorigenic signaling but also contributes to the disruption of the intestinal epithelial barrier, thereby increasing intestinal permeability and promoting microbial translocation. Impairment of epithelial barrier integrity is considered a critical link between microbial dysbiosis and chronic inflammation, and its maintenance depends on the coordinated function of the mucus layer, tight junctions, and adherens junctions [23]. Under inflammatory and tumor-associated conditions, expression levels of tight junction proteins such as ZO-1, occludin, and claudin-3, as well as the adherens junction protein E-cadherin, are markedly reduced, resulting in widened intercellular spaces and increased epithelial permeability [33, 34]. In addition, abnormalities in the MUC2 gene may lead to thinning of the mucus layer, rendering the intestinal epithelium more susceptible to microbial and inflammatory stimuli, which is considered an early event in inflammation initiation [24].

Inflammatory cytokines and aberrant signaling pathways are actively involved in barrier disruption. TNF-α and IFN-γ significantly reduce transepithelial electrical resistance (TEER) and disrupt tight junction architecture through upregulation of the uPA-uPAR system [34]. P2RY13 has also been shown to promote epithelial apoptosis and tight junction degradation through activation of the IL-6/STAT3 signaling pathway [35]. Moreover, IL-1β upregulates myosin light-chain kinase (MLCK) via NF-κB and p38 MAPK signaling, thereby promoting dissociation of tight junction complexes. IL-1β may additionally enhance epithelial permeability through miR-200c-3p-mediated degradation of occludin [36].

A bidirectional relationship exists between microbial dysbiosis and epithelial barrier dysfunction. ETBF weakens epithelial adhesion by cleaving E-cadherin, whereas F. nucleatum enhances epithelial permeability and facilitates bacterial penetration of the epithelial barrier through its FadA protein [30, 37]. Meanwhile, dysbiosis-associated metabolic alterations may further impair barrier protection; for example, butyrate deficiency induces claudin-2 upregulation and promotes bacterial translocation [24]. Deficiency of NOD1 and NOD2 accelerates epithelial apoptosis and increases permeability, thereby enhancing susceptibility to inflammation-associated tumorigenesis [23, 28].

Once barrier integrity is compromised, luminal bacteria and their products, including lipopolysaccharide (LPS), can penetrate the epithelium and enter the lamina propria and systemic circulation, resulting in microbial translocation that persistently activates innate immune responses and amplifies inflammatory signaling [32, 33]. Dietary emulsifiers and high-saturated-fat diets may also promote inflammation-associated lesions by altering microbial composition and impairing barrier stability [27].

#### Microbiota-driven inflammatory responses and immune activation

Barrier disruption and microbial translocation further promote local immune activation in the intestine, driving the transition from transient host defense responses to persistent chronic inflammation. Microbial components are recognized by pattern recognition receptors such as TLR4 and NOD receptors, leading to activation of inflammatory signaling pathways such as NF-κB and STAT3, accompanied by increased production of pro-inflammatory cytokines, including TNF-α, IL-6, and IL-1β, as well as impaired anti-inflammatory regulation mediated by IL-10, thereby establishing a sustained pro-inflammatory microenvironment [29, 33, 35]

Microbiota-derived metabolites also play pivotal roles in maintaining immune homeostasis. Secondary bile acid metabolites such as 3-oxoLCA suppress Th17 differentiation, whereas isoalloLCA promotes regulatory T cell (Treg) development. Imbalances in these metabolites contribute to dysregulation of the Th17/Treg axis and further exacerbate inflammation [24, 31]. In addition, tryptophan metabolites suppress pro-inflammatory cytokine release and maintain mucosal immune homeostasis through activation of AhR signaling, whereas disruption of this pathway contributes to persistent mucosal inflammation [28, 38].

Prolonged inflammatory stimulation further contributes to the establishment of a tumor-promoting immune microenvironment. Chronic activation of Th17-related inflammatory responses and impaired antitumor immunity facilitate epithelial injury and immune dysregulation during colitis-associated carcinogenesis. In addition, pathogenic bacteria such as F. nucleatum can suppress the cytotoxic activities of T cells and natural killer (NK) cells while promoting M2 macrophage polarization, thereby favoring immune evasion and tumor progression [28, 29, 37].

**Fig. 1:**
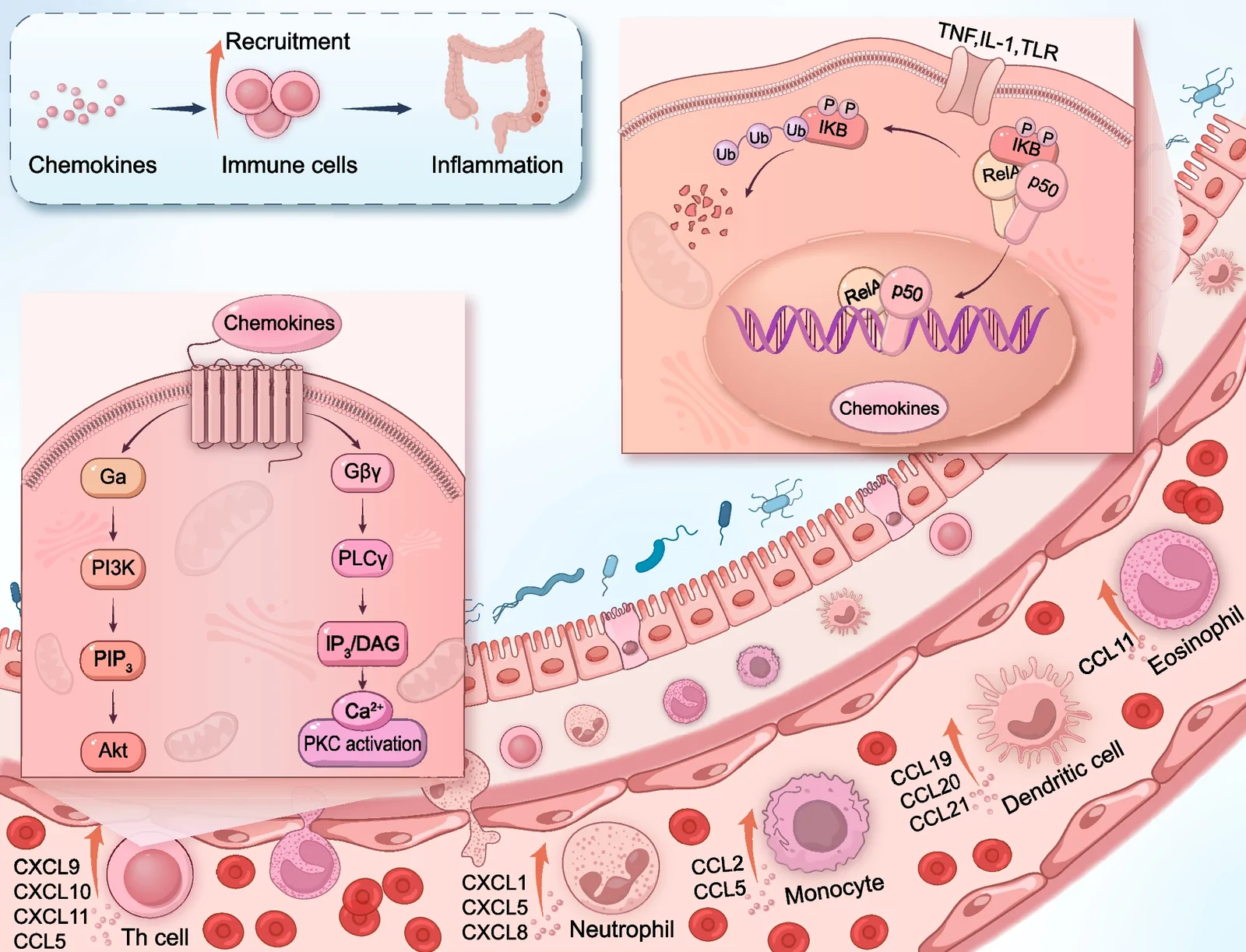
Chemokine-mediated inflammatory signaling and immune-cell recruitment in the intestinal microenvironment. The schematic illustrates a cross-section of the colonic mucosa and summarizes the central axis “chemokines → immune cells → inflammation”. Inflammatory stimuli such as TNF-α, IL-1, and dysbiosis activate NF-κB signaling, upregulating multiple chemokines (e.g., CXCL8, CCL2). These upstream signals originate from cytokine receptors and pattern-recognition receptors such as Toll-like receptors (TLRs), leading to intracellular activation of NF-κB and transcription of chemokine genes. Distinct chemokine subfamilies engage CXCR and CCR receptors to direct the recruitment and activation of immune cells, thereby establishing a positive inflammatory feedback loop that perpetuates chronic inflammation and tissue damage and ultimately promotes inflammation-associated tumorigenesis. For example, chemokines such as CXCL9, CXCL10, CXCL11, and CCL5 recruit Th cells, whereas CXCL8 and CCL2 attract neutrophils and monocytes/macrophages to sites of intestinal inflammation. Upon binding to their receptors on immune cells, chemokines activate downstream signaling pathways involving heterotrimeric G proteins. The dissociated Gα and Gβγ subunits subsequently stimulate intracellular effectors such as phospholipase C (PLC) and phosphatidylinositol-3-kinase (PI3K), which regulate calcium signaling, protein kinase activation, and cytoskeletal rearrangement required for leukocyte chemotaxis. Abbreviations: IKK, IκB kinase; IκB, inhibitor of NF-κB; RelA, NF-κB p65 subunit; Ub, ubiquitin; Gα, G-protein alpha subunit; PI3K, phosphatidylinositol-3-kinase; PIP3, phosphatidylinositol (3,4,5)-trisphosphate; PLC, phospholipase C

Chronic inflammation, in turn, favors the continued expansion of pathogenic bacteria and further disruption of microbial homeostasis, forming a persistent positive feedback loop involving microbial dysbiosis, barrier disruption, and immune activation [32]. During this process, multiple chemokines become aberrantly upregulated and participate in immune cell recruitment, amplification of inflammation, and remodeling of the TME, suggesting that chemokines may serve as critical regulators linking chronic inflammation to colorectal tumorigenesis (Fig. 1).

### Chemokine-mediated inflammatory amplification and immune cell recruitment

#### Dysregulated chemokine expression in inflammatory colorectal microenvironments

In response to persistent inflammatory stimulation, dysregulated chemokine networks become major drivers of immune cell recruitment and inflammatory amplification during inflammation-associated colorectal carcinogenesis. Chemokines are a family of small chemotactic cytokines that regulate immune-cell trafficking and inflammatory responses through interactions with G protein-coupled receptors (GPCRs) [39–41]. In chronic inflammation and TME, dysregulation of chemokine expression promotes persistent recruitment of immune cells, exacerbation of the inflammatory response, and tissue remodeling; therefore, chemokine signaling pathways represent potential therapeutic targets for CRC and other inflammatory diseases [42]. Chemokines are broadly classified into four subfamilies (CXC, CC, C, and CX3C), among which the CC and CXC families are most prominently involved in intestinal inflammation and inflammation-associated colorectal carcinogenesis [43] (Table 1).

**Table 1:** Classification and functions of chemokines

Chemokine	Receptor	Function	References
CCL2	CCR2	Promotes recruitment of inflammatory cells (e.g., monocytes, macrophages), maintains an immunosuppressive microenvironment, and facilitates intestinal carcinogenesis	[44]
CCL3	CCR1, CCR5	Initiates inflammatory responses and promotes intestinal tumor progression and metastasis	[45]
CCL5	CCR5	Recruits immunosuppressive T cells and MDSCs, enhancing tumor immune evasion	[46]
CCL20	CCR6	Recruits Th17 cells, amplifies local inflammation, and promotes intestinal tumor development	[47]
CCL22	CCR4	Induces Treg chemotaxis, suppresses antitumor immunity, and promotes immunosuppression within TMEs	[48]
CXCL1	CXCR2	Promotes neutrophil infiltration, enhances local inflammation, and synergizes in tumor promotion	[49]
CXCL8	CXCR1, CXCR2	Stimulates neutrophil and macrophage chemotaxis, promoting intestinal inflammation and tumor progression	[50]
CXCL12	CXCR4	Facilitates cancer cell proliferation and metastasis, and induces immune evasion within TMEs	[51]

During the progression of IBD, chemokine expression displays distinct disease-associated patterns. In the intestinal mucosa of patients with active UC or CD, levels of chemokines such as CXCL8, CXCL10, and CCL2 are significantly elevated, correlating positively with disease activity and inflammation severity [52, 53]. Remarkably, chemokine expression patterns differ between UC and CD. CXCL8 exhibits diffuse expression in the colonic mucosa of UC patients but focal expression in CD, mainly produced by neutrophils, macrophages, and intestinal epithelial cells [54]. This distinction reflects their distinct pathological features. UC primarily involves continuous mucosal inflammation, whereas CD exhibits transmural, segmental, and discontinuous lesions [55, 56]. These differences are linked not only to genetic, microbial, and environmental factors [57], but also to dysregulated chemokine signaling networks that shape local immune responses. Overexpression of the CXC family (e.g., CXCL8, CXCL10) and CC family (e.g., CCL2) is common in IBD and contributes to sustained immune cell recruitment and chronic inflammation, thereby establishing a persistent inflammatory milieu that predisposes the colon to subsequent tumor development [58, 59].

#### Upstream regulation and downstream signaling pathways of chemokines

At the upstream regulatory level, chemokine production and receptor expression are controlled by multiple signaling pathways. On the one hand, chemokine expression is mainly regulated by signaling pathways and transcription factors such as p38 MAPK and NF-κB. In addition, intestinal epithelial cells can secrete chemokines to participate in immune defense under infectious stimulation [60, 61] (Fig. 1). On the other hand, cytokines such as IL-2 in T cells can regulate the expression of chemokine receptors. Th1 and Th2 subsets selectively express characteristic receptors. In addition, the expression of certain intestinal mucosa–related receptors (such as CCR6 and CCR9) is tissue-specific [62, 63].

At the downstream signal transduction level, the binding of chemokines to their receptors activates G proteins, whose dissociated subunits further trigger key enzymes including phospholipase C (PLC) and phosphatidylinositol 3-kinase (PI3K) [64, 65]. PLC regulates intracellular Ca^2+^ levels and protein kinase activity (e.g., PKC) [66], while the PI3K p110γ isoform governs leukocyte survival and migration [67]. Together, these pathways mediate immune cell chemotaxis and activation, forming the molecular foundation for chronic intestinal inflammatory cascades and contributing to the establishment of tumor-promoting inflammatory microenvironments [39] (Fig. 1). Importantly, activation of NF-κB and PI3K-related signaling pathways has also been confirmed in colonic biopsy samples from patients with active UC and CD, supporting the clinical relevance of these mechanisms beyond experimental models. These findings indicate that core chemokine-mediated signaling cascades are conserved between DSS-induced models and human IBD, and may contribute directly to inflammation-associated colorectal carcinogenesis [68, 69].

#### Functional roles of major chemokine axes in inflammatory and tumor-promoting microenvironments

In the pathogenesis of colitis, the CXC and CC subfamilies play central roles in coordinating immune-cell recruitment and inflammatory amplification. Their contributions to tumor progression through microenvironment remodeling are discussed in later sections [70, 71]. The CXC family can be categorized into ELR⁺ and ELR⁻ types according to the presence of a glutamate-leucine-arginine (ELR) motif. ELR⁺ chemokines primarily target neutrophils, whereas ELR⁻ members are involved in Th1-type immune response [70, 72]. The CC subfamily exhibits broader immunomodulatory functions by recruiting monocytes/macrophages, lymphocytes, dendritic cells, and eosinophils, thereby shaping the immune-cell composition of the TME [39, 73].

##### ELR⁺ CXC chemokines recruit neutrophils to drive inflammation

CXCL8 (IL-8) serves as a central ELR⁺ chemokine, with its expression correlating with disease activity. It is diffusely expressed in UC and focally in CD, mainly secreted by neutrophils, macrophages, and epithelial cells, recruiting neutrophils to sites of inflammation, and has been implicated in promoting tumor cell proliferation, angiogenesis, and metastatic potential in CRC [54, 74, 75]. CXCL1 of the same family promotes both neutrophil recruitment and angiogenesis, serving as a potential biomarker for colitis, and may further contribute to tumor-promoting angiogenesis and microenvironment remodeling [74]. CXCL5 (ENA-78), negatively correlated with disease activity, mediates sustained granulocyte recruitment in late inflammation and synergizes with CXCL8 to enhance chemotaxis, which may support chronic inflammatory niches that favor tumor initiation and progression [76] (Fig. 1).

##### ELR⁻ CXC chemokines recruit and activate lymphocytes to drive Th1-type inflammation

In Th1-dominant IBD, including CD, CXCL9, CXCL10, and CXCL11 are markedly upregulated in the intestinal mucosa, and are associated with immune activation patterns observed in inflammation-driven tumorigenesis [77]. Immunohistochemical analyses of intestinal biopsy specimens from patients with CD have demonstrated increased CXCR3 expression on mucosal T lymphocytes, supporting its role in recruiting Th1 effector cells to inflamed intestinal tissues, which may contribute to persistent immune activation in tumor-prone environments [78, 79]. In addition, elevated levels of CXCR3 ligands such as CXCL9 and CXCL11 have been associated with disease activity and recurrence in clinical cohorts [80, 81]. By binding to their common receptor CXCR3, they selectively recruit CXCR3⁺Th1 cells to inflamed sites [82]. These Th1 cells further secrete IFN-γ and TNF-α [83], activating macrophages and enhancing antigen presentation [84], thereby establishing a Th1-polarized microenvironment that exacerbates mucosal injury and chronic inflammation, conditions that are closely linked to inflammation-associated tumor initiation [85, 86]. Furthermore, CXCL10 (IP-10) expression is also elevated in UC, but is more prominent in CD, correlating with Th1 immune intensity, and may influence tumor immune surveillance and progression depending on the inflammatory context [87] (Fig. 1).

##### CC chemokines recruit target cells via specific receptors to drive inflammation and immune responses

CCL2 expression is elevated in the inflamed mucosa of patients with UC and CD, as demonstrated by colonic biopsy analyses [88, 89], where it primarily recruits monocytes and macrophages, contributing to the accumulation of TAMs in CRC [90]. Its role is particularly prominent in colorectal involvement of CD and is regulated by proinflammatory cytokines such as TNF-α [89, 91]. CCL5 is induced under combined TNF-α and IFN-γ stimulation [92], efficiently recruiting T lymphocytes and monocytes/macrophages to inflamed tissue, and may enhance immune cell-mediated modulation of the TME [93]. CCL11 (Eotaxin-1), a potent eosinophil chemoattractant, is mainly secreted by cells including macrophages and epithelial tuft cells [94]. Its expression is regulated by IFN-γ [95], and it functions primarily via the CCR3 receptor, with additional binding to CCR2, CCR5, and CXCR3. It is constitutively expressed in the gastrointestinal tract and contributes to colitis by recruiting eosinophils, an activity dependent on its species-specific NH₂-terminal 25 amino acid, which may also participate in shaping local immune responses in tumor-associated inflammation [96]. Elevated CCL11 expression has also been detected in intestinal biopsy samples from patients with active UC, where eosinophil infiltration parallels local chemokine expression levels [94]. Additionally, CCL19, CCL21, and CCL20 play pivotal roles in dendritic cell (DC) trafficking. CCL19 and CCL21, through CCR7, guide mature DCs to T-cell zones in lymphoid organs, which is a key step in initiating adaptive immune responses relevant to both inflammation and tumor immunity [97, 98]. Meanwhile, CCL20 via CCR6 specifically recruits mucosal DC subsets in intestinal barrier tissues, mediating immune surveillance and inflammation, and has been implicated in regulating immune cell infiltration in colorectal tumor tissues [99] (Fig. 1). In patients with IBD, increased mucosal expression of CCL20 and CCR6 has been observed in biopsy specimens, particularly in areas with active epithelial damage, suggesting conserved mechanisms between human disease and experimental colitis models, which may extend to inflammation-associated colorectal carcinogenesis [100, 101].

Although many chemokines amplify inflammatory cascades, their functions are highly context-dependent. Certain chemokines, such as CXCL10 and CX3CL1, can enhance protective immune surveillance by recruiting cytotoxic T cells or NK cells during early inflammatory responses, which may exert antitumor effects in early disease stages [102–104]. In addition, several regulatory mechanisms help restrain excessive chemokine activity. For example, receptor desensitization and internalization can attenuate sustained signaling [105, 106], while atypical chemokine receptors (ACKRs), such as ACKR2, act as chemokine scavengers to remove inflammatory ligands and limit leukocyte over-recruitment, thereby preventing excessive inflammation and potentially limiting tumor-promoting conditions [107, 108]. Therefore, chemokine networks in CAC are not exclusively pro-inflammatory but are dynamically regulated depending on disease stage, immune context, and the balance between inflammatory and regulatory signals.

Collectively, these interconnected factors sustain chronic intestinal inflammation and create a pro-tumorigenic microenvironment. Persistent inflammatory stimuli are subsequently translated into aberrant intracellular signaling, contributing to epithelial dysfunction, genomic instability, and malignant transformation.

## Molecular mechanisms of CAC

### Inflammatory signaling pathways

Unlike sporadic CRC, which is primarily driven by early genetic alterations through the adenoma–carcinoma sequence, CAC arises within a background of chronic inflammation, where persistent inflammatory signaling acts as a primary driver of subsequent molecular alterations and malignant transformation [109, 110]. This sustained signaling activates multiple inflammatory and oncogenic pathways, including TNF-α/NF-κB, IL-6/JAK/STAT3, COX-2/PGE2, PI3K/AKT/mTOR, and Wnt/β-catenin, thereby promoting epithelial survival and proliferation and facilitating malignant transformation [18, 111, 112]. For instance, STAT3 can be activated by multiple ligands and plays a critical role in regulating cell cycle progression and apoptosis. Sustained activation of STAT3 target genes further promotes cell growth, angiogenesis, and cell motility while inhibiting apoptosis, thereby facilitating tumor progression [113–115]. Collectively, the interaction between chemokines and inflammatory signaling pathways establishes a positive regulatory network that sustains inflammation and contributes to tumor development.

#### TNF-α/NF-κB signaling sustains chronic inflammation

The TNF-α/NF-κB signaling pathway is a pivotal inflammatory signaling axis driving the initiation and progression of CAC [14, 116, 117]. During chronic colitis, persistent inflammatory stimulation results in sustained activation of NF-κB in intestinal epithelial cells and immune cells, promoting epithelial proliferation, resistance to apoptosis, and production of pro-inflammatory mediators that facilitate tumor initiation and progression [118–120].

**Fig. 2:**
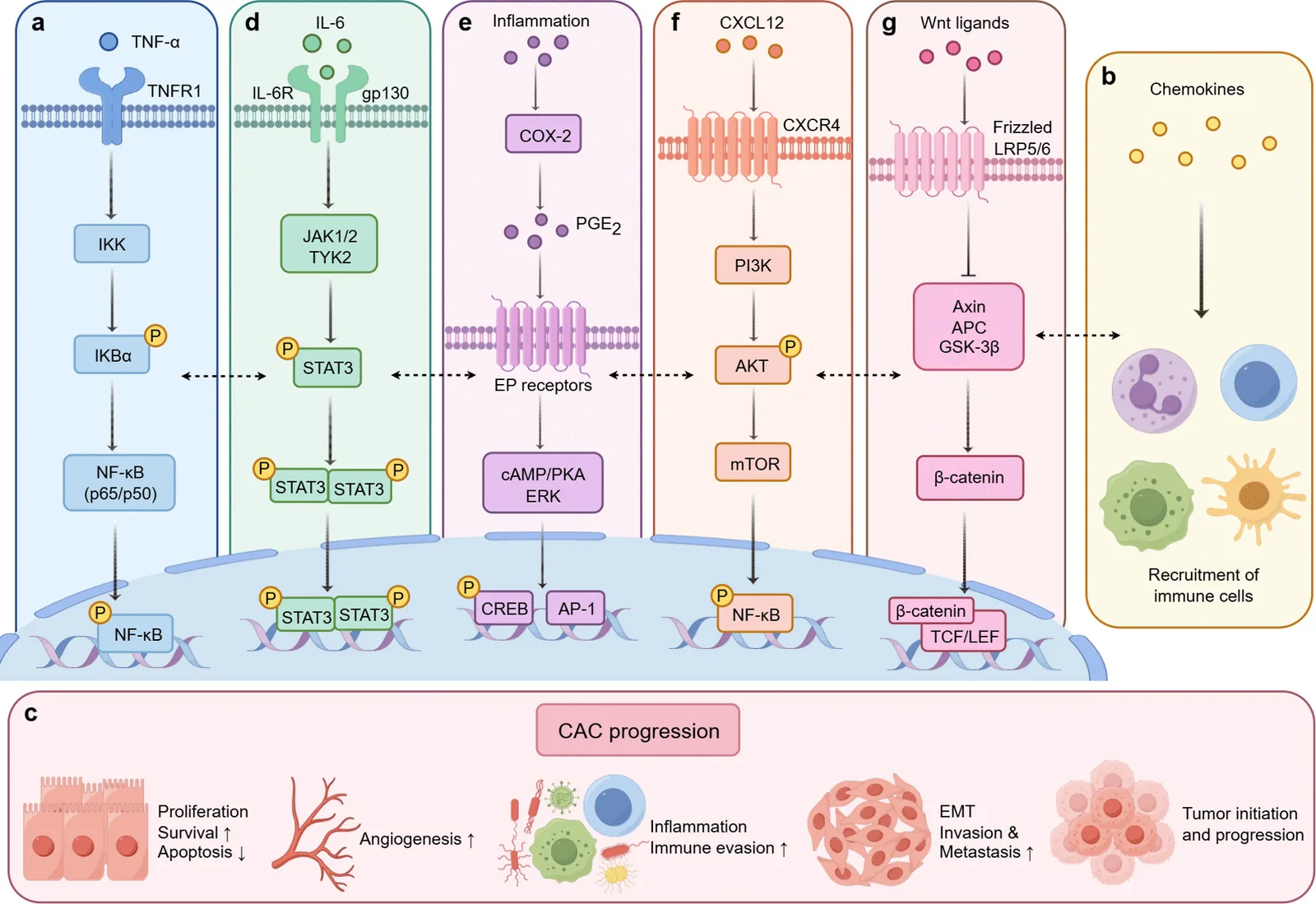
Interconnected inflammatory and oncogenic signaling pathways in CAC progression. This schematic illustrates the major inflammatory and oncogenic signaling pathways involved in colitis-associated colorectal carcinogenesis (CAC) and their interactions with chemokine networks. **a** TNF-α binding to TNFR1 activates the IKK complex, leading to IκBα phosphorylation and subsequent NF-κB activation, which promotes the transcription of genes involved in inflammation, cell survival, angiogenesis, and proliferation. **b** Chemokine networks are interconnected with these inflammatory and oncogenic signaling pathways and contribute to the recruitment of immune cells, thereby linking inflammatory signaling with immune regulation in the CAC microenvironment. **c** The integrated activation of these interconnected pathways contributes to CAC progression by promoting epithelial proliferation and survival, suppressing apoptosis, enhancing angiogenesis, sustaining inflammation and immune evasion, and facilitating EMT, invasion, metastasis, and tumor initiation and progression. **d** IL-6 binding to IL-6R/gp130 activates JAK1/2 and TYK2, resulting in STAT3 phosphorylation, dimerization, and nuclear translocation to regulate genes associated with cell proliferation, survival, angiogenesis, and immune regulation. **e** Inflammatory stimuli induce COX-2 and increase PGE2 production, which activates EP receptors and downstream cAMP/PKA and ERK signaling to promote epithelial proliferation, survival, angiogenesis, and immune suppression. **f** The CXCL12/CXCR4 axis activates PI3K/AKT/mTOR signaling, enhancing cell proliferation, survival, migration, invasion, EMT, and angiogenesis. **g** Wnt ligands activate Frizzled/LRP5/6 receptors and inhibit the Axin/APC/GSK-3β destruction complex, resulting in β-catenin stabilization and nuclear translocation, where β-catenin/TCF/LEF signaling promotes epithelial proliferation and stemness. Abbreviations: COX-2, cyclooxygenase-2; CXCL12, C-X-C motif chemokine ligand 12; CXCR4, C-X-C motif chemokine receptor 4; EMT, epithelial–mesenchymal transition; PGE2, prostaglandin E2; TCF/LEF, T-cell factor/lymphoid enhancer factor

TNF-α is one of the major upstream activators of NF-κB [121]. Binding of TNF-α to TNFR1 recruits adaptor proteins and activates the IκB kinase (IKK) complex, leading to phosphorylation and degradation of IκBα [122, 123]. This process releases the NF-κB p65/p50 heterodimer, allowing its nuclear translocation and transcriptional activation of genes involved in inflammation, cell survival, angiogenesis, and proliferation, including IL-6, COX-2, BCL-XL, and Cyclin D1 [124–126] (Fig. 2a).

IL-1β concurrently amplifies this process. Following inflammasome activation, mature IL-1β binds to IL-1R and activates the MyD88-dependent signaling pathway, resulting in additional NF-κB activation [127–129]. Elevated IL-1β levels contribute to persistent inflammatory responses, epithelial regeneration, and tumor-promoting microenvironment formation in CAC [130, 131].

Toll-like receptors (TLRs) provide another important upstream mechanism for NF-κB activation [132]. Disruption of the intestinal epithelial barrier during colitis increases exposure to microbial pathogen-associated molecular patterns, particularly lipopolysaccharide (LPS), which activates TLR4-mediated signaling [133, 134]. Through MyD88-dependent pathways, TLR activation stimulates the IKK complex and sustains NF-κB activation, establishing a chronic inflammatory microenvironment favorable for tumor development [135, 136].

It is important to note that chemokines can serve both as downstream targets of the NF-κB signaling pathway and as indirect amplifiers of it [137, 138]. NF-κB transcriptionally upregulates multiple chemokines, including CXCL1, CXCL8, CCL2, and CCL20, which recruit neutrophils, monocytes, and immunosuppressive cells into the intestinal mucosa [139] (Fig. 2b). These infiltrating immune cells produce additional TNF-α and IL-1β, forming a positive feedback loop that perpetuates NF-κB activation and accelerates CAC progression [140] (Fig. 2c).

#### IL-6/JAK/STAT3 signaling promotes tumor progression

The IL-6/JAK/STAT3 signaling pathway is another key inflammatory axis linking chronic colitis to colorectal carcinogenesis [141]. Persistent inflammation in the intestinal mucosa results in sustained production of interleukin-6 (IL-6) by macrophages, fibroblasts, and intestinal epithelial cells, leading to activation of STAT3 during CAC development [142].

IL-6 exerts its biological effects by binding to IL-6R, followed by association with gp130 [143, 144]. This interaction activates Janus kinases (JAK1, JAK2, and TYK2), which phosphorylate STAT3 [145, 146]. Phosphorylated STAT3 dimerizes and translocates into the nucleus, where it induces the transcription of genes involved in cell proliferation, survival, angiogenesis, and immune regulation [147]. Persistent STAT3 activation therefore promotes epithelial hyperproliferation, inhibits apoptosis, and facilitates malignant transformation during chronic intestinal inflammation (Fig. 2d).

Beyond its direct effects on tumor cells, STAT3 also contributes to the establishment of an immunosuppressive tumor microenvironment [148]. Activated STAT3 stimulates the production of inflammatory mediators and promotes the expansion of MDSCs, TAMs, and Tregs, thereby sustaining chronic inflammation and facilitating immune evasion [149, 150]. Furthermore, the NF-κB and STAT3 pathways can mutually activate each other, thereby amplifying inflammatory signal transduction and accelerating the progression of CAC [151].

Importantly, chemokines further activate STAT3 signaling [152]. Several chemokines, including CXCL1, CXCL8, CXCL12, and CCL2, activate STAT3 through their receptors in tumor cells and stromal cells, thereby enhancing tumor cell proliferation, angiogenesis, epithelial–mesenchymal transition (EMT), and metastatic potential [153, 154] (Fig. 2b). These interactions establish a positive feedback network between inflammatory cytokines, chemokines, and STAT3, contributing to the initiation and progression of CAC (Fig. 2c).

#### COX-2/PGE2 signaling promotes tumor growth and immune suppression

The COX-2/PGE2 signaling pathway is one of the classical inflammatory mechanisms underlying CAC [155]. During chronic intestinal inflammation, COX-2 is induced by pro-inflammatory cytokines, microbial products, and oxidative stress, resulting in increased production of PGE2 [155–157].

PGE2 exerts its biological effects through EP receptors, activating downstream pathways [158] (Fig. 2e). These signaling events promote epithelial cell proliferation, inhibit apoptosis, stimulate angiogenesis, and facilitate EMT, thereby accelerating the progression from chronic colitis to CRC [159, 160] (Fig. 2c). In addition, PGE2 contributes to an immunosuppressive tumor microenvironment by impairing anti-tumor immune responses [161].

The COX-2/PGE2 pathway also exhibits extensive crosstalk with NF-κB and STAT3 signaling [159, 162]. Persistent activation of these pathways further enhances COX-2 expression, forming a positive feedback loop that sustains chronic inflammation and tumor progression [163, 164]. Moreover, chemokines interact closely with the COX-2/PGE2 axis, promoting inflammatory cell recruitment and reinforcing the pro-tumorigenic inflammatory microenvironment during CAC development [165] (Fig. 2b).

#### PI3K/AKT/mTOR signaling promotes cell survival and metabolic reprogramming

The PI3K/AKT/mTOR signaling pathway is a key regulator of cell growth, proliferation, metabolism, and survival, and its aberrant activation is frequently observed during CAC [166, 167]. Chronic inflammation promotes PI3K activation through inflammatory cytokines and growth factors, resulting in phosphorylation of AKT and subsequent activation of mTOR [168]. Persistent activation of this pathway enhances intestinal epithelial cell proliferation, inhibits apoptosis, and promotes protein synthesis and metabolic reprogramming, thereby facilitating malignant transformation [166].

Among the upstream regulators, the CXCL12/CXCR4 axis is one of the best-characterized chemokine pathways activating PI3K/AKT/mTOR signaling in CAC [169]. Binding of CXCL12 to CXCR4 stimulates PI3K activation, leading to AKT phosphorylation and mTOR signaling [170]. This cascade promotes tumor cell proliferation, migration, invasion, and survival while also enhancing EMT and angiogenesis. In addition, CXCL12/CXCR4 signaling interacts with Wnt/β-catenin and NF-κB, further accelerating colorectal tumor progression [169, 171]. Therefore, the CXCL12/CXCR4–PI3K/AKT/mTOR axis represents an important molecular link between chronic inflammation and colorectal carcinogenesis and may serve as a promising therapeutic target in CAC (Fig. 2f).

#### Wnt/β-catenin signaling promotes epithelial proliferation and stemness

The Wnt/β-catenin signaling pathway is a fundamental regulator of intestinal epithelial homeostasis and is frequently dysregulated during CAC [172, 173]. Under physiological conditions, β-catenin is continuously degraded by the APC/Axin/GSK-3β destruction complex [174]. Upon activation of Wnt signaling, this degradation complex is inhibited, allowing β-catenin to accumulate in the cytoplasm and translocate into the nucleus, where it interacts with TCF/LEF transcription factors to induce the expression of genes involved in cell proliferation and survival [175–177].

Chronic intestinal inflammation contributes to persistent activation of Wnt/β-catenin signaling through inflammatory cytokines and oxidative stress, thereby promoting uncontrolled epithelial proliferation, inhibiting apoptosis, and facilitating malignant transformation [178, 179]. Increasing evidence also suggests extensive crosstalk between the Wnt/β-catenin pathway and NF-κB, STAT3, and PI3K/AKT, which together drive CAC progression [12] (Fig. 2g).

Importantly, chemokines further promote Wnt/β-catenin signaling [180]. Chemokine axes such as CXCL12/CXCR4 enhance β-catenin stabilization and nuclear translocation through activation of PI3K/AKT and other downstream pathways, thereby promoting tumor cell proliferation, stemness, migration, and invasion [169, 181]. These findings indicate that chemokines not only regulate inflammatory cell recruitment but also reinforce oncogenic Wnt signaling, contributing to the initiation and progression of CAC [180] (Fig. 2b).

Collectively, these signaling pathways do not function independently but form an interconnected inflammatory network sustained by reciprocal interactions among inflammatory cytokines, chemokines, immune cells, and intestinal epithelial cells [154, 155]. Persistent activation of the TNF-α/NF-κB, IL-6/JAK/STAT3, COX-2/PGE₂, PI3K/AKT/mTOR, and Wnt/β-catenin pathways establishes a self-amplifying pro-tumorigenic microenvironment that represents a defining molecular characteristic of CAC [182] (Fig. 2c).

### Oxidative stress and DNA damage

#### Mitochondrial dysfunction exacerbates DNA damage by promoting endogenous ROS production

In the chronic inflammatory microenvironment, mitochondrial function of epithelial cells is continuously impaired, manifested by abnormal electron transport chains and decreased membrane potential, resulting in electron leakage and the production of a large number of reactive oxygen species (mtROS) derived from mitochondria [183]. Excessive mtROS not only directly oxidize DNA bases but also induce both single- and double-strand DNA breaks and compromise the base excision repair (BER) system [184, 185]. In addition, mitochondrial damage can also release mitochondrial DNA (mtDNA) into the cytosol as damage-associated molecular patterns (DAMPs) to further activate the inflammatory response [186]. These processes collectively promote DNA damage accumulation and genomic instability, enhance tumorigenesis risk, and play a key role in the positive inflammatory-oxidative stress feedback loop.

#### Chemokines amplify oxidative stress by recruiting immune cells, thereby sustaining inflammation

**Fig. 3:**
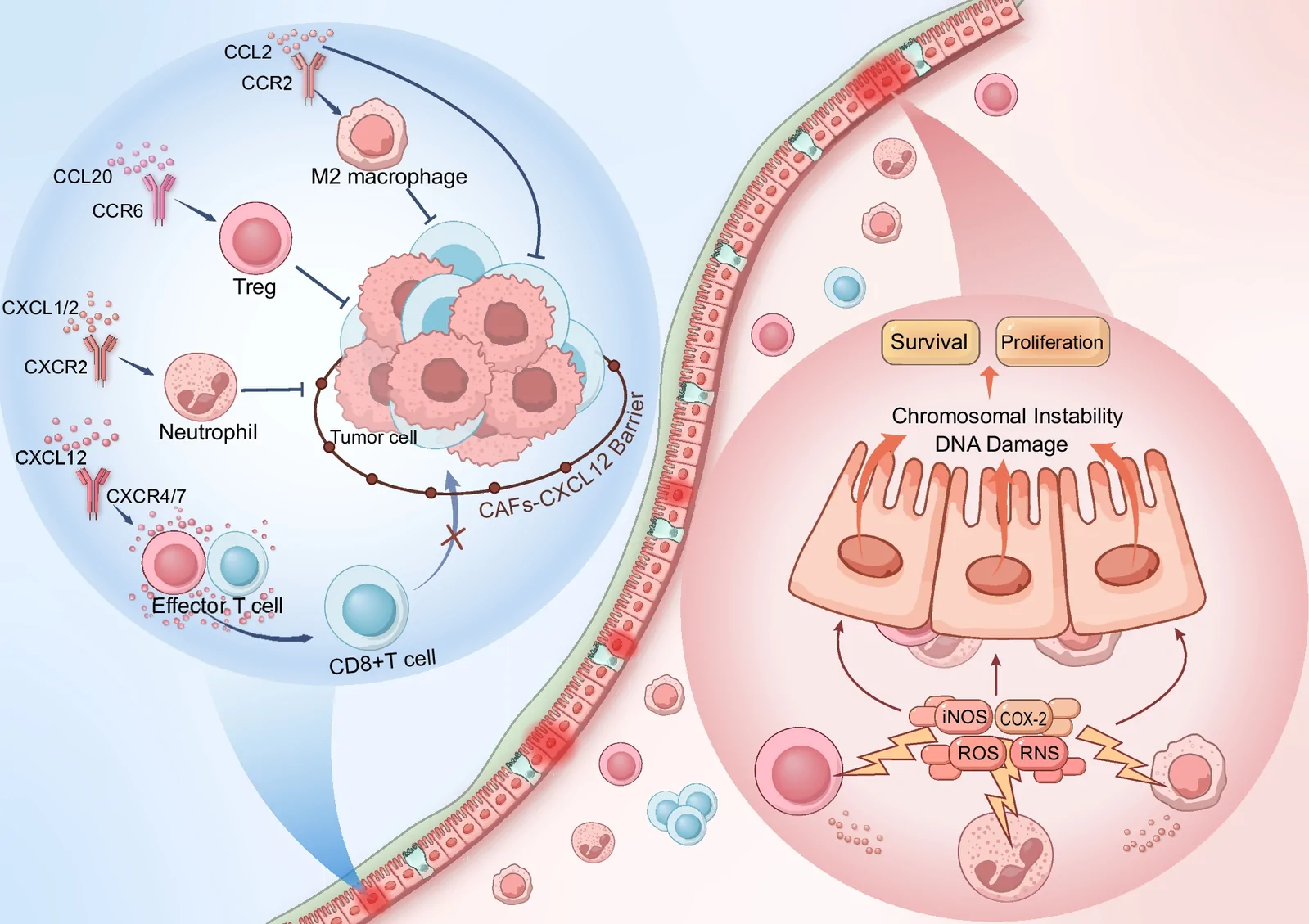
Chemokines recruit immunosuppressive cells to mediate inflammatory cancer transformation. This schematic illustrates how chemokine networks reshape the tumor immune microenvironment during the transition from chronic inflammation to CRC. Under persistent inflammatory conditions, intestinal epithelial cells, stromal cells, and cancer-associated fibroblasts secrete multiple chemokines that recruit immunosuppressive immune cells to the TME, thereby weakening immune surveillance and promoting tumor progression. Multiple chemokine axes are involved in this process. The CCL2–CCR2 axis recruits circulating monocytes to tumor sites, where they differentiate into TAMs. These TAMs secrete immunosuppressive and tumor-promoting factors such as IL-10, TGF-β, ARG1, and VEGF, which suppress cytotoxic T-cell activity and promote angiogenesis and epithelial–mesenchymal transition. The CCL20–CCR6 axis mediates the recruitment of regulatory Tregs into the TME, where Tregs further suppress CD8^+^ T-cell and dendritic-cell functions through cytokines including IL-10 and TGF-β. In addition, the CXCL1/2–CXCR2 axis promotes the recruitment of PMN-MDSCs, which inhibit antitumor immune responses and facilitate tumor growth. The CXCL12–CXCR4 axis contributes to immune-exclusion mechanisms that prevent effective infiltration of effector T cells into the TME. Abbreviations: TME, tumor microenvironment; TAMs, tumor-associated macrophages; PMN-MDSCs, polymorphonuclear myeloid-derived suppressor cells

Chemokines attract immune cells such as neutrophils and macrophages to the mucosa, where they become activated and generate substantial amounts of reactive oxygen species (ROS) and proinflammatory enzymes (e.g., iNOS, COX-2). These mediators cause DNA damage, base excision repair errors, and chromosomal instability in neighboring epithelial cells, thereby increasing the likelihood of oncogene activation and tumor suppressor gene inactivation. This process sustains inflammation and promotes tumorigenesis [3, 12, 187, 188] (Fig. 3).

#### Imbalance of gut microbiota induces oxidative stress and DNA damage through metabolites

Imbalance of intestinal flora is one of the important triggers for CAC. Pathogenic bacteria can produce a variety of pro-oxidant metabolites, such as hydrogen sulfide, secondary bile acids, and bacterial toxins, which can directly induce epithelial cells to produce ROS and damage DNA structures [189, 190]. In addition, some bacteria (such as toxic Escherichia coli) can secrete genotoxins that directly trigger DNA double-strand breaks [191]. The imbalance of the flora can also disrupt the intestinal mucosal barrier, promote the infiltration of inflammatory cells and amplify the inflammatory response, thus forming a vicious circle of "bacterial dysfunction-oxidative stress-DNA damage" and accelerating tumorigenesis [192].

#### The role of NADPH oxidase system activation in oxidative stress amplification

The NADPH oxidase (NOX) family is an important source of ROS in inflammatory conditions. In colitis, the expression of NOX1, NOX2 and NOX4 is upregulated and continuously activated, resulting in the production of superoxide anions and hydrogen peroxide in large quantities [193]. These ROS not only directly induce oxidative damage to DNA, but also further enhance inflammatory responses and cell proliferation by activating downstream signaling pathways such as MAPK and NF-κB [194]. At the same time, NOX-mediated ROS production has an "amplification effect", which can stimulate the recruitment and activation of more immune cells, thereby maintaining a state of high oxidative stress and promoting the accumulation of DNA damage and tumor progression [195]. Accumulated oxidative damage and chronic inflammatory stress further drive genetic and epigenetic alterations, which cooperate with chemokine signaling to sustain tumor-promoting programs. Unlike sporadic CRC, where microsatellite instability (MSI) most commonly results from primary mismatch repair deficiency, genomic instability in CAC is largely driven by persistent inflammatory injury and oxidative DNA damage [196, 197]. Although MSI is detected in a subset of CAC cases, chromosomal instability and inflammation-induced mutagenesis are generally considered the predominant forms of genomic instability in CAC [109].

### Genetic and epigenetic alterations

Persistent oxidative stress and impaired DNA repair not only promote mutation accumulation but also reshape the genetic and epigenetic landscape of CAC [8, 11]. Consequently, CAC follows a molecular evolutionary trajectory distinct from sporadic CRC, which typically progresses through the conventional adenoma–carcinoma sequence. Under persistent inflammatory stress [9, 198], the timing and spectrum of genetic and epigenetic alterations in CAC are profoundly reshaped.

#### Chemokines cooperate with oncogene activation and tumor suppressor gene loss to drive malignant transformation

**Fig. 4:**
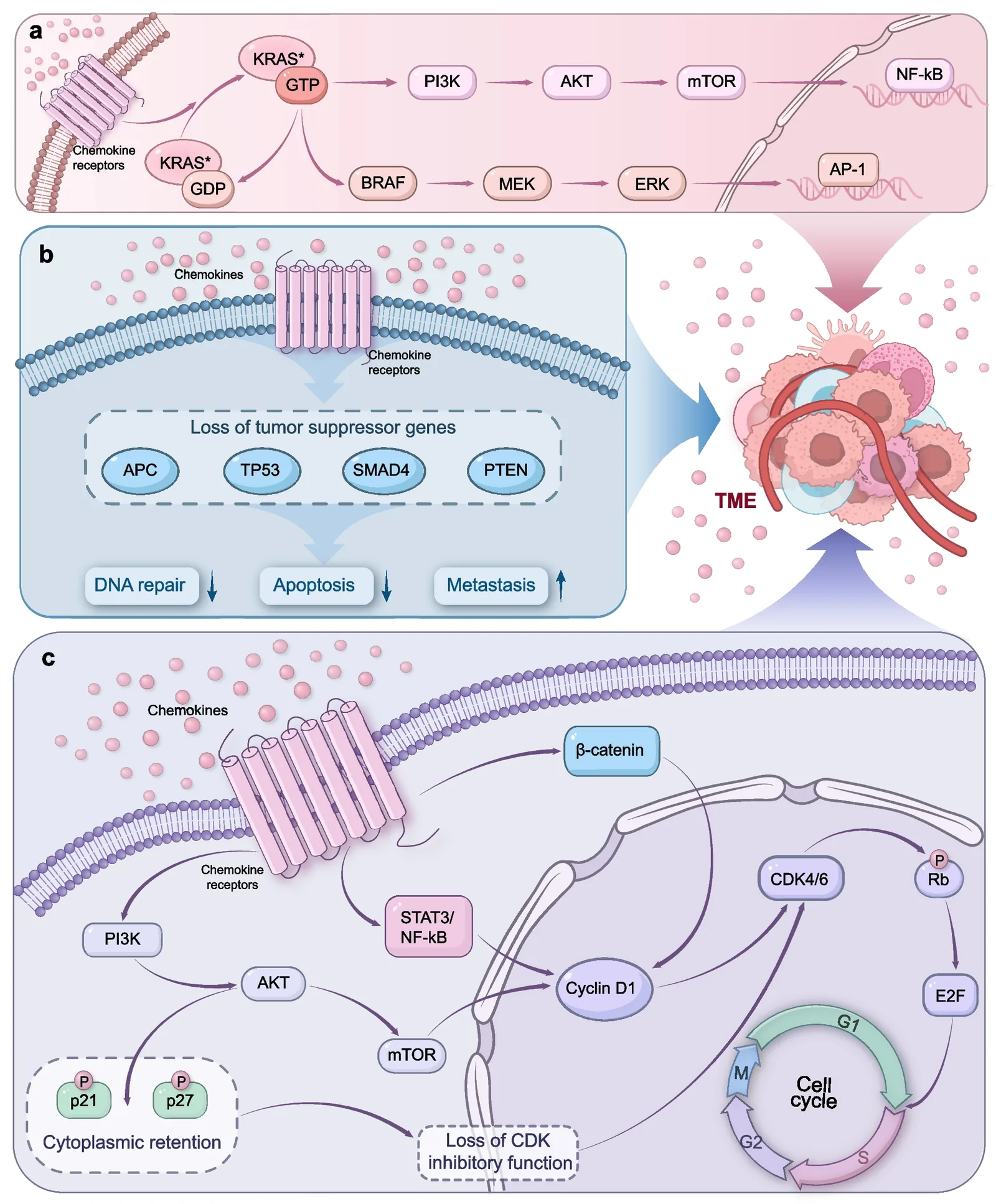
Chemokine-driven integration of oncogenic signaling and cell-cycle dysregulation in colorectal carcinogenesis. **a** Chemokines activate oncogenic signaling networks and form positive feedback loops with mutated proto-oncogenes. In CRC, constitutively active KRAS enhances downstream RAF–MEK–ERK and PI3K–AKT–mTOR pathways, leading to activation of transcription factors such as AP-1 and NF-κB. This promotes tumor cell proliferation, survival, invasion, and inflammation, while simultaneously inducing the secretion of chemokines and cytokines that reinforce a pro-tumorigenic microenvironment. **b** Chemokine signaling synergizes with the loss of tumor suppressor genes (TSGs). Activation of STAT3 and NF-κB pathways downstream of chemokine receptors cooperates with the inactivation of key TSGs (e.g., APC, TP53, PTEN, SMAD4), resulting in impaired DNA damage repair, reduced apoptosis, enhanced cell survival, and increased invasion and metastasis, thereby driving malignant transformation and tumor progression. **c** Chemokines disrupt cell-cycle homeostasis through coordinated regulation of cyclins and CDK inhibitors. Chemokine-mediated activation of β-catenin and STAT3/NF-κB signaling induces Cyclin D1 expression, which promotes CDK4/6 activation, Rb phosphorylation, and E2F release, accelerating G1/S transition. Concurrently, activation of the PI3K–AKT pathway induces phosphorylation of p21 and p27, causing their cytoplasmic retention and loss of CDK-inhibitory function. Together, these mechanisms remove cell-cycle checkpoints and promote uncontrolled cell proliferation. Abbreviations: KRAS, Kirsten rat sarcoma viral oncogene homolog; GTP, Guanosine triphosphate; BRAF, B-Raf proto-oncogene; RAF, Rapidly Accelerated Fibrosarcoma kinase; MEK, MAPK/ERK kinase; ERK, Extracellular signal-regulated kinase; AP-1, Activator protein-1; NF-κB, Nuclear factor kappa-light-chain-enhancer of activated B cells; STAT3, Signal transducer and activator of transcription 3; APC, Adenomatous polyposis coli; TP53, Tumor protein p53; PTEN, Phosphatase and tensin homolog

Although CAC and sporadic CRC share several oncogenic pathways, the molecular evolution of CAC is strongly influenced by chronic inflammation. Proto-oncogenes such as KRAS, BRAF, and PI3K are cellular genes expressed during normal growth and development [199, 200]. Alterations in these genes are closely associated with the development of human malignancies [201, 202]. However, proto-oncogenes do not function independently; rather, they operate within complex signaling networks that are influenced by chemokine-mediated signaling [203]. For instance, in CRC, KRAS mutations impair its intrinsic GTPase. Impaired GTP hydrolysis activity induces constitutive activation, which not only autonomously drives tumor cell proliferation but also boosts secretion of various chemokines such as CXCL1 and CXCL3 [204–206]. Many of these factors, in turn, may further contribute to the establishment of an immunosuppressive environment that supports tumor progression [207] (Fig. 4a).

#### Chemokines synergize with the loss of tumor suppressor gene function

Cancer development is generally considered to result from the accumulation of genetic and epigenetic alterations affecting oncogenes and tumor suppressor genes (TSGs). Mutations in proto-oncogenes are typically dominant in nature, converting them into oncogenes, whereas TSGs represent another essential category involved in DNA damage repair, inhibition of cell division, induction of apoptosis, and suppression of metastasis. Consequently, the loss of TSG function contributes to cancer initiation and progression [208]. However, during CRC development, the chemokine signaling network does not act independently of TSG loss; instead, it engages in intricate synergistic and antagonistic interactions [209–211] (Fig. 4b). Compared with sporadic CRC, which commonly follows the adenoma–carcinoma sequence initiated by APC mutation, CAC exhibits a distinct order of molecular alterations [212]. TP53 mutation and chromosomal abnormalities frequently occur at earlier stages of CAC, whereas APC mutations are generally considered later events during malignant progression [213, 214]. These differences highlight the unique evolutionary trajectory of inflammation-associated colorectal carcinogenesis and suggest that chronic inflammatory stress influences the selection of genetic alterations.

#### Chemokines disrupt cell cycle homeostasis to promote tumorigenesis

The progression of the cell cycle is driven by the periodic expression and activation of cyclins and cyclin-dependent kinases (CDKs), while CDK inhibitors (CKIs) exert negative regulatory control. However, chemokines disrupt this delicate balance through multiple mechanisms [215, 216]. Directly, they activate signaling pathways that modulate Cyclin D1 and CKIs. Indirectly, they recruit immunosuppressive cells (TAMs, MDSCs, Tregs) into the TME [217]. These cells secrete a variety of immunosuppressive and pro-tumorigenic mediators, including IL-10, TGF-β, ARG1, and VEGF. IL-10 and TGF-β suppress antitumor immune responses, ARG1 impairs T-cell function through arginine depletion, and VEGF promotes tumor angiogenesis. Collectively, these factors enhance epithelial cell proliferation and survival, thereby facilitating tumorigenesis and disease progression. Chemokine-mediated disruption of cell-cycle homeostasis can be broadly divided into induction of Cyclin D1 expression and suppression of CKIs.

Induction of Cyclin D1 expression occurs when chemokine-mediated nuclear translocation of β-catenin directly upregulates Cyclin D1 [180, 216]. In addition, the STAT3/NF-κB signaling axis transcriptionally enhances Cyclin D1 expression, promoting the G1/S transition [218]. The PI3K–Akt–mTOR pathway stabilizes Cyclin D1 and simultaneously enhances CDK4/6 activity, promoting Rb phosphorylation and E2F release. As a result, cell cycle progression into the S phase is accelerated [219] (Fig. 4c).

Suppression of CKIs involves Akt-mediated phosphorylation of p21 and p27, resulting in their cytoplasmic retention and loss of nuclear CDK-inhibitory function, effectively releasing the G1/S checkpoint brake. This represents another crucial node through which inflammatory chemokine signaling modulates the cell cycle via the PI3K–Akt pathway [220, 221] (Fig. 4c).

#### Chronic inflammation induces aberrant DNA methylation through TET repression and DNMT activation

Chronic inflammation disrupts DNA methylation homeostasis through two coordinated mechanisms: suppression of TET-mediated DNA demethylation and enhancement of DNMT activity.

Pro-inflammatory cytokines such as TNF-α and IL-1β activate NF-κB signaling, thereby inducing inflammation-associated miRNAs that repress TET family proteins. As key dioxygenases responsible for the conversion of 5-methylcytosine to 5-hydroxymethylcytosine, reduced TET activity impairs DNA demethylation capacity. ChIP-seq analyses further demonstrate enhanced binding of the NF-κB subunit RELA to the promoter regions of MIR26B (CTDSP1) and MIR20A (MIR17HG). Consistently, TET downregulation has been observed in multiple models of chronic inflammation [222].

Concurrently, nitric oxide (NO) produced by NOS2 enhances DNMT enzymatic activity, further promoting aberrant methylation. Notably, TET repression and DNMT activation act synergistically; knockdown of TET3 alone or NO exposure alone induces aberrant methylation only in a minimal number of genomic blocks, whereas their combination markedly expands the methylated regions [222].

Unlike sporadic CRC, in which the CpG island methylator phenotype (CIMP) represents a well-recognized molecular subtype characterized by widespread promoter hypermethylation, epigenetic alterations in CAC are thought to arise predominantly as a consequence of chronic inflammation [11, 223]. In CAC, promoter hypermethylation represents a major mechanism for tumor suppressor gene inactivation, affecting genes such as MLH1 (a DNA mismatch repair gene), CDKN2A (encoding the cell cycle inhibitor p16INK4a), and APC (a negative regulator of WNT signaling). In the colonic mucosa of patients with long-standing UC, methylation of p14ARF has been detected as an early event prior to the appearance of dysplasia [14].

In parallel with regional hypermethylation, global hypomethylation is also a hallmark of IBD and CAC, particularly affecting repetitive elements such as LINE-1. LINE-1 hypomethylation is associated with poor clinical outcomes and may contribute to genomic instability. The coexistence of regional hypermethylation and global hypomethylation constitutes a characteristic epigenetic pattern in inflammation-associated carcinogenesis [14].

#### Non-coding RNA networks in colitis-associated carcinogenesis

In addition to DNA methylation changes, chronic inflammation also reshapes non-coding RNA regulatory networks that directly modulate chemokine production and signaling.

##### NF-κB-dependent and STAT3–miR-21 regulatory circuits that sustain chemokine production

NF-κB-dependent microRNAs play critical roles in modulating inflammatory signaling networks. Among them, miR-146a and miR-155 represent opposing yet complementary regulators of NF-κB signaling. miR-146a is rapidly induced by NF-κB and directly targets TRAF6 and IRAK1, forming a classical negative feedback loop that restrains NF-κB activation [224]. In contrast, miR-155 targets negative regulators such as SOCS1 and SHIP1, thereby amplifying JAK/STAT and NF-κB signaling pathways. In the context of chronic inflammation, sustained upregulation of these miRNAs may disrupt the balance between negative and positive feedback regulation, converting physiological control mechanisms into a pathological circuit that perpetuates NF-κB and STAT3 activation. As a result, transcription of multiple chemokines (e.g., CXCL8 and CCL2) is continuously enhanced, contributing to persistent inflammatory signaling [14].

miR-21 is one of the most prominently upregulated oncogenic miRNAs in both IBD and CRC. IL-6–activated STAT3 directly binds to the miR-21 promoter and induces its transcription. miR-21 subsequently suppresses PTEN and CYLD, negative regulators of the PI3K/AKT and NF-κB pathways, respectively. PTEN inhibition sustains AKT activation, while CYLD inhibition enhances NF-κB signaling, collectively promoting proliferation, survival, and inflammatory signaling. This establishes a positive feedback loop in which IL-6/STAT3 signaling induces miR-21, and miR-21 in turn reinforces both NF-κB and AKT pathways, further sustaining STAT3 activation. Even transient activation of this circuit has been shown to promote cellular transformation [225]. In CRC tissues, elevated miR-21 expression correlates with downregulation of PDCD4 and enhanced pro-inflammatory and pro-invasive signaling [225]. Sustained miR-21 expression not only promotes epithelial cell proliferation and survival but also indirectly enhances transcription of multiple chemokines through relief of NF-κB inhibition, thereby reshaping the TME.

##### Direct regulation of chemokine axes by miRNAs

In addition to indirect regulation through signaling pathways, specific miRNAs can directly control chemokine expression at the post-transcriptional level. miR-141 directly targets the 3′ UTR of CXCL12 and suppresses its expression. Downregulation of miR-141 in CRC leads to CXCL12 overexpression, thereby enhancing tumor cell migration and metastasis through the CXCL12/CXCR4 axis. Restoration of miR-141 expression significantly inhibits this invasive phenotype [226], highlighting a direct mechanistic link between miRNA dysregulation and chemokine network rewiring during colitis-associated carcinogenesis.

Taken together, chemokine signaling cooperates with genetic alterations and integrates cell cycle dysregulation with aberrant DNA methylation and miRNA network rewiring in colitis-associated carcinogenesis [109]. These molecular alterations differ from those observed in sporadic CRC by evolving within a background of persistent inflammation, thereby reinforcing the concept that CAC represents a distinct inflammation-driven subtype of CRC. By disrupting cell cycle checkpoints, inducing DNA methylation alterations, and reshaping miRNA networks, these mechanisms collectively silence tumor suppressor pathways and sustain pro-inflammatory signaling. These alterations form a self-reinforcing network in which persistent chemokine production, epigenetic instability, and inflammatory signaling mutually amplify each other, ultimately driving the progression from chronic colitis to carcinoma. Notably, the regulatory effects of these epigenetic circuits are context-dependent [224]. Collectively, these findings indicate that chronic inflammation reshapes the genetic and epigenetic evolution of CAC, generating molecular features that distinguish it from sporadic CRC. Beyond intrinsic alterations in epithelial cells, chronic inflammation and chemokine dysregulation also remodel the TME, thereby establishing immunosuppressive and pro-metastatic niches that facilitate CAC progression.

### TME remodeling and immune dysregulation

#### Chronic inflammation reshapes the immunosuppressive tumor microenvironment

Persistent intestinal inflammation promotes the accumulation of immunosuppressive cells within the TME. CRC-bearing mice exhibit elevated levels of MDSCs, which localize near invading bacteria and acquire enhanced suppressive functions [227]. TAMs frequently polarize toward an M2-like phenotype, characterized by secretion of IL-10, TGF-β, and VEGF, thereby suppressing cytotoxic T cell activity and promoting angiogenesis [228].

In addition to myeloid-cell remodeling, chronic inflammatory stress also alters Treg metabolic adaptation and effector function within the TME. In the glucose-restricted CRC microenvironment, Tregs downregulate the MondoA–TXNIP axis, leading to enhanced glycolysis and IL-17A production, which subsequently drives CD8⁺ T-cell exhaustion [229]. CAFs further reinforce immunosuppression through stromal remodeling and inflammatory signaling. Deletion of IL-1R2 reduces tumor burden and enhances exhausted CD8⁺ T cell responses, indicating that IL-1R2 signaling in Tregs and myeloid cells sustains an immunosuppressive niche [230].

Collectively, persistent inflammation drives coordinated interactions among immune cells, stromal cells, and cytokines, establishing a tolerogenic microenvironment that facilitates immune evasion and malignant progression.

#### Metabolic reprogramming and hypoxia drive immune dysfunction

Hypoxia and metabolic reprogramming are key drivers of immune dysfunction in CRC. Hypoxia-associated molecular subtyping identifies a C1 subtype with high levels of immune exclusion and increased infiltration of exhausted CD8⁺ T cells, including terminal Tex and OXPHOS-Tex, which are linked to poor immunotherapy response [231].

Metabolic byproduct accumulation further exacerbates immune suppression within the TME. Lactate accumulation is a hallmark of the Warburg effect. Tumor-derived lactate upregulates histone lactylation, driving NUPR1 expression in TAMs; NUPR1 promotes M2 polarization and PD-L1 expression via ERK/JNK inhibition, inducing CD8⁺ T cell exhaustion [232]. Oxidative stress and lipid metabolic reprogramming further impair antitumor immunity by promoting inflammasome activation and CD8⁺ T-cell exhaustion [233].

Collectively, hypoxia, metabolic reprogramming, and metabolite accumulation cooperatively reshape the immune landscape, establishing a metabolically restrictive and immunosuppressive microenvironment that sustains chronic inflammation and malignant progression.

#### Chemokine networks remodel the immunosuppressive tumor microenvironment

Beyond their established roles in inflammatory cell recruitment during chronic colitis (Section “ Chemokine-mediated inflammatory amplification and immune cell recruitment”), chemokines actively remodel the TME after malignant transformation by orchestrating the functional polarization, spatial organization, and immunosuppressive activities of multiple stromal and immune cell populations [234, 235]. Through these effects, chemokine networks promote immune evasion and sustain tumor progression.

The CCL2–CCR2 axis contributes to an immunosuppressive tumor microenvironment by promoting the accumulation and functional polarization of TAMs through IL-6/STAT3 and NF-κB signaling [236, 237]. These TAMs produce IL-10, TGF-β, ARG1, and VEGF, thereby suppressing CD8^+^ T-cell activity and promoting angiogenesis, EMT, and tumor progression [238, 239] (Fig. 3). CCL2 signaling may also reinforce myeloid-cell-mediated immunosuppression through ROS production and STAT3 activation [238, 240]. Consistent with these findings, elevated CCL2 expression is associated with macrophage infiltration and disease progression in human CRC tissues [241].

Similarly, the CCL20–CCR6 axis contributes to local immune suppression by promoting Treg accumulation and M2-like macrophage activity. Treg-derived IL-10 and TGF-β suppress CD8⁺ T-cell and dendritic-cell functions, whereas M2-like macrophages further reinforce an immunosuppressive state [242–245] (Fig. 3). The positive association between CCL20 expression and FOXP3⁺ Treg infiltration in human CRC tissues supports the clinical relevance of this pathway [243].

The CXCL1/2–CXCR2 axis further sustains immunosuppression through MDSC accumulation and impaired CD8⁺ T-cell function [246–249] (Fig. 3). Increased CXCR2 expression is associated with infiltration of MDSCs and neutrophil-like myeloid cells and correlates with tumor progression and poor survival in CRC patients [249, 250].

In addition, CAF-derived CXCL12 contributes to stromal remodeling and immune exclusion through the CXCL12–CXCR4/7 axis, limiting the infiltration of effector T cells into tumor cores [251–254] (Fig. 3). Elevated CXCL12/CXCR4 expression in human CRC tissues is associated with tumor metastasis and poor clinical outcomes [255, 256].

These molecular alterations contribute to CAC initiation and progression, while the resulting changes in the intestinal mucosa, tumor microenvironment, and circulating molecular profiles may also provide clinically relevant signals for early detection and disease surveillance.

## Diagnostic and surveillance strategies

### Molecular biomarkers associated with CAC progression and surveillance

#### CXCR4 and CCL20 as potential biomarkers associated with tumor progression and inflammatory activity

CXCR4 is highly expressed in over 70% of CRC tissues, particularly in stages III–IV. Meta-analysis shows high sensitivity (92%) but limited specificity (53%) for poor prognosis [254]. This limited specificity may reflect its broad upregulation in various malignancies and inflammatory conditions, suggesting that CXCR4 alone is not a highly specific diagnostic indicator. Instead, it may be more valuable as a marker of tumor aggressiveness or microenvironmental activation. CCL20 is elevated in Fn-high CRC tissues, positively correlating with Fn abundance (r = 0.3882, P = 0.0004), indicating potential as a microbial-driven CRC biomarker [245]. These findings suggest that chemokine-associated biomarkers may not only reflect tumor aggressiveness but also capture the persistent inflammatory characteristics underlying CAC progression (Table 2).

**Table 2:** Diagnostic biomarkers and surveillance strategies for CAC

Specific biomarker/method	Associated background/mechanism	Potential clinical application	References
CXCR4	High in > 70% CRC (stages III–IV); high sensitivity (92%) but low specificity (53%)	Marker of tumor aggressiveness/microenvironment activation	[254]
CCL20	Elevated in F. nucleatum-high CRC, positively correlated with Fn abundance	Microbial-driven CRC biomarker	[245]
CCL8	High in TAMs of Fn-positive/high-iron CRC; linked to shorter OS in stage I–III	Prognostic biomarker for that subgroup	[257]
Exosomal miR-146a-5p & miR-155-5p	Released by CXCR7-high CRC cells, activate CAFs via JAK2–STAT3/NF-κB, promote metastasis	Non-invasive circulating biomarkers for metastasis (needs validation)	[258]
KRT17–CXCL10 axis	In dMMR CRC, high KRT17 stabilizes CXCL10, increasing CD8⁺ T-cell infiltration	Predictor of anti-PD-1 response in dMMR CRC	[259]
Chromoendoscopy-guided targeted biopsy	Enhances visualization of subtle, flat, and multifocal dysplastic lesions; improves targeted biopsy accuracy	Improved dysplasia detection and biopsy accuracy	[260–262]
SCENIC consensus	Recommends chromoendoscopy with targeted biopsies and lesion classification	Standardized surveillance and management	[260, 262]
AI-assisted endoscopic surveillance	Improves detection/characterization of subtle lesions, reduces variability	Enhanced detection, risk stratification, personalized monitoring when integrated with molecular biomarkers	[263–265]

#### CCL8 as a prognostic biomarker in inflammation and microbiota-associated CRC subgroups

In CRC patients with Fn positivity and high serum iron (TSAT ≥ 30%), the prognosis is significantly worse. Increased CCL8 expression in TAMs has been linked to poor prognosis in this high-risk subgroup. TCGA analysis links high CCL8 with shorter OS in stage I–III CRC (P = 0.0077). These results suggest that CCL8 can serve as a potential prognostic biomarker for Fn-positive, high-iron CRC patients [257]. However, its prognostic value in other subgroups requires further study (Table 2).

#### Exosomal miRNAs and circulating biomarkers for dynamic disease monitoring

CRC cells with high expression of CXCR7 release miR-146a-5p and miR-155-5p via exosomes, activating the JAK2–STAT3/NF-κB pathway in CAFs and promoting tumor metastasis. Clinical validation showed that the levels of these exosomal miRNAs were markedly increased in the serum of metastatic CRC patients and positively correlated with CXCR7 expression. These findings indicate that these miRNAs could serve as non-invasive circulating biomarkers of CXCR7 activity and metastatic progression [258]. However, prospective validation in larger cohorts is still required to determine their clinical utility for dynamic metastasis monitoring (Table 2).

#### KRT17–CXCL10 axis as a predictor of immunotherapy response in dMMR CRC

In mismatch repair-deficient (dMMR) CRC, high expression of keratin 17 (KRT17) inhibits YTHDF2-mediated CXCL10 mRNA degradation, promoting elevated CXCL10 levels, which in turn recruit more CD8⁺ T cells to infiltrate TME. Clinical validation indicates that patients with high KRT17 expression have better responses to anti-PD-1 therapy, with predictive efficacy superior to traditional assessments of CD8⁺ T-cell density. Therefore, the KRT17–CXCL10 axis holds promise as a novel biomarker for predicting immunotherapy response in dMMR CRC [259]. Clinical relevance depends on molecular subtype and immune context (Table 2).

Thus, individual chemokines are unlikely to serve as universal CRC biomarkers due to context-dependent expression; combinatorial panels may offer greater specificity but require prospective validation and standardized detection methods.

### Chromoendoscopy-guided biopsy: current standard surveillance approach for CAC

Regular colonoscopic surveillance is recommended for patients with long-standing IBD due to their increased risk of developing CAC [260, 266]. However, inflammation-related mucosal changes and the subtle morphology of dysplastic lesions make early detection challenging [3, 267]. Chromoendoscopy, using topical contrast agents such as indigo carmine or methylene blue, enhances mucosal visualization and improves the identification of subtle, flat, and multifocal dysplastic lesions compared with conventional white-light endoscopy [261, 268].

Current surveillance strategies emphasize targeted biopsy of suspicious lesions identified by chromoendoscopy rather than extensive random biopsies [260, 269]. By improving lesion recognition and biopsy accuracy, chromoendoscopy-guided targeted biopsy increases diagnostic yield and facilitates early intervention for dysplasia, representing a key approach in CAC surveillance (Table 2) [261, 262].

### Current consensus guidelines for IBD-associated colorectal neoplasia surveillance: SCENIC and beyond

The SCENIC consensus provides an important framework for surveillance and management of IBD-associated colorectal neoplasia, including CAC, in patients with long-standing IBD [260]. The consensus recommends high-quality colonoscopic surveillance with chromoendoscopy-guided targeted biopsies as the preferred approach for detecting dysplasia, as this strategy improves visualization of subtle mucosal abnormalities and enhances diagnostic accuracy compared with conventional random biopsy protocols [260, 262].

Furthermore, SCENIC emphasizes the importance of standardized management of visible dysplastic lesions by distinguishing between polypoid and non-polypoid lesions [260]. This classification assists clinical decision-making regarding endoscopic resection or surgical intervention, particularly colectomy, according to lesion characteristics, resectability, and underlying disease context [260]. Despite these advances, challenges remain in optimizing surveillance intervals and accurately identifying high-risk patients, highlighting the need for emerging technologies such as AI-assisted endoscopy (Table 2).

### Artificial intelligence-assisted endoscopic surveillance in CAC

Although colonoscopic surveillance with targeted biopsy remains the cornerstone for detecting dysplasia in patients with long-standing IBD, accurate identification of early neoplastic lesions remains challenging due to subtle morphological changes and inter-observer variability [3, 260, 270]. Recent advances in artificial intelligence-assisted endoscopy have further improved dysplasia detection and lesion characterization in IBD surveillance [264, 271].

AI-assisted endoscopy can enhance the detection and characterization of dysplastic lesions by analyzing real-time endoscopic images and recognizing subtle abnormalities, including small, flat, and non-polypoid lesions that may be difficult to identify by conventional endoscopy [263, 265]. These systems may reduce diagnostic variability among endoscopists, improve targeted biopsy accuracy, and increase the efficiency of dysplasia surveillance [263, 265].

Beyond lesion detection, AI-based models integrating endoscopic features with clinical characteristics, imaging data, and molecular biomarkers may further improve risk stratification and early identification of high-risk IBD patients [264, 272, 273]. Therefore, the combination of AI-assisted endoscopy with molecular biomarkers and standardized surveillance strategies represents a promising direction for personalized CAC monitoring (Table 2).

Although advances in biomarkers and surveillance strategies have improved the identification of high-risk individuals, these approaches alone cannot prevent disease progression or eliminate the underlying drivers of carcinogenesis. Effective prevention and treatment therefore remain essential for reducing CAC incidence and progression.

## Preventive and therapeutic approaches

### Chemopreventive strategies

Several classical anti-inflammatory agents exert preventive effects by inducing broad but context-dependent suppression of chemokine expression. High-dose 5-aminosalicylic acid (5-ASA, ≥ 1.2 g/day) inhibits NF-κB signaling, leading to reduced expression of multiple inflammatory mediators and chemokines, including IL-6, TNF-α, and MCP-1 [274]. A meta-analysis revealed that long-term 5-ASA administration reduces the risk of colorectal tumor development by 49% (OR = 0.51) [275, 276]. Oral administration of docosahexaenoic acid (DHA, 10 mg/kg) inhibited the STAT3–ERK signaling pathway, thereby reducing MCP-1 levels by 55%, macrophage infiltration by 42%, and tumor count by 72% [277]. These findings suggest that suppression of inflammatory signaling and chemokine production may contribute to limiting tumor development (Table 3).

**Table 3:** Preclinical and clinical studies of major therapeutic strategies for CAC

Intervention strategy	Clinical trial identifier & status	Mechanism of action and key findings	References
5-ASA	NCT04920149 (recruiting); NCT03070574 (terminated)	Inhibit NF-κB signaling, reduce IL-6, TNF-α, and MCP-1 levels, and lower CRC risk in IBD patients	[274–276]
DHA	NCT00024401 (status unknown; last known status: active, not recruiting, 2002)	Suppress STAT3/ERK signaling, reduce MCP-1 expression and macrophage infiltration, and inhibit CAC development	[277]
Non-peptide CCR2/CCR5/CXCR3 antagonist	Not applicable (preclinical only)	Block inflammatory cell recruitment, attenuate colitis, and suppress inflammation-driven tumorigenesis	[278]
CXCR4 antagonist (e.g., AMD3100)	NCT02179970 (completed)	Inhibit inflammatory cell migration and immunosuppressive microenvironment formation; alleviate colitis and reduce tumorigenesis	[74, 279]
Propagermanium	Not applicable (preclinical only)	Reduce macrophage infiltration, downregulate COX-2, and inhibit angiogenesis, thereby suppressing CAC progression	[280]
SP600125 (JNK inhibitor)	Not applicable (preclinical only)	Inhibit JNK signaling and reduce CXCL1 expression, suppressing aberrant epithelial proliferation	[235]
RS504393 (CCR2 inhibitor)	Not applicable (preclinical only)	Inhibit CCR2 signaling, attenuate pro-tumor inflammation, and suppress CAC progression	[281]
CCR2⁻/⁻ bone marrow chimeras	Not applicable (genetically modified animal model)	Validate the pathogenic role of CCR2 signaling in inflammation-associated colorectal tumorigenesis	[281]
Anti-CXCL1 neutralizing antibody	Not applicable (preclinical only)	Inhibit MDSC and TAM recruitment, reducing tumor number and burden	[235]
Anti-CCL2 neutralizing antibody	Not applicable (preclinical only)	Block monocyte/macrophage recruitment and limit immunosuppressive microenvironment formation	[238]
CCL2 N-terminal deletion mutant (7ND)	Not applicable (preclinical only)	Antagonize CCL2 signaling and suppress inflammatory cell infiltration and tumor formation	[282]
AAV8-SARI gene therapy	Not applicable (preclinical only)	Downregulate MCP-1 expression and suppress tumorigenesis	[281]
PLGA nanoparticle co-delivery of SARI and CCR2-siRNA	Not applicable (preclinical only)	Enhance inhibition of MCP-1/CCR2 signaling and improve anti-inflammatory efficacy	[281]
CXCR3 inhibitor + PD-1 inhibitor	Not applicable (preclinical only)	Reduce Treg-mediated immunosuppression and enhance immunotherapeutic efficacy	[283, 284]
Oral sodium tungstate	Not applicable (preclinical only)	Suppress pathogenic Enterobacteriaceae expansion and reduce CAC incidence	[285]

### Molecularly targeted and immune-modulatory strategies

Unlike conventional anti-inflammatory interventions, molecularly targeted strategies aim to directly interfere with specific chemokine axes, inflammatory signaling pathways, or immune regulatory mechanisms involved in CAC initiation and progression. These approaches provide improved mechanistic specificity; however, most current evidence remains derived from preclinical models, and their clinical applicability in CAC requires further validation.

#### Chemokine axis blockade

Chemokine signaling plays a central role in regulating inflammatory cell recruitment, immune suppression, and tumor-promoting microenvironment formation during CAC development. Therefore, direct blockade of chemokines or their receptors represents an attractive therapeutic strategy.

Small-molecule antagonists targeting multiple chemokine receptors have demonstrated protective effects in experimental colitis models. A non-peptide small molecule capable of simultaneously antagonizing CCR2, CCR5, and CXCR3 alleviated DSS-induced colitis, suggesting that simultaneous modulation of multiple chemokine axes may be required to effectively control intestinal inflammation [278]. Similarly, CXCR4 antagonists significantly attenuated DSS-induced and IL-10⁻/⁻-mediated colitis, supporting the CXCL12/CXCR4 axis as an important therapeutic target for early intervention [74, 279].

Beyond receptor blockade, selective inhibition of specific chemokines has also shown therapeutic potential. Propagermanium, a CCR2 antagonist, suppresses the CCL2/CCR2 signaling axis, reduces macrophage infiltration, downregulates COX-2 expression, and inhibits angiogenesis in CAC models [280]. Furthermore, anti-CXCL1 neutralizing antibodies effectively block the RIP3–CXCL1 axis, leading to reduced tumor number, size, and burden in CAC mice. This effect is accompanied by decreased infiltration of MDSCs and M2-like TAMs and enhanced recruitment of T cells into the tumor microenvironment [235] (Table 3).

Similarly, inhibition of CCL2 signaling represents another promising approach. Anti-CCL2 neutralizing antibodies reduce PMN-MDSC accumulation, ROS production, and PD-L1 expression in tumors, while blocking the transition from dysplasia to adenocarcinoma in CAC models. Mechanistically, these effects are mediated through inhibition of CCL2-driven STAT3 activation and suppression of immunosuppressive myeloid cell function [238]. In addition, the CCL2 antagonist 7ND reduces macrophage infiltration, decreases COX-2 expression, inhibits angiogenesis, and suppresses tumor development [282].

Nevertheless, given the complexity and redundancy of chemokine networks, long-term blockade of individual chemokine pathways may require careful evaluation regarding efficacy, toxicity, and compensatory pathway activation.

#### Inflammatory signaling inhibition and pathway modulation

Because chemokine expression is tightly regulated by inflammatory signaling networks, targeting upstream pathways represents another strategy to prevent inflammation-driven carcinogenesis.

The IL-6/JAK/STAT3 signaling pathway is a critical regulator of chronic inflammation and tumor-promoting immune responses in CAC. Tofacitinib, by inhibiting JAK1/3, blocks the IL-6/STAT3 feedback loop, reducing the secretion of downstream chemokines such as MCP-1 and IL-6 [286, 287]. A phase II clinical trial demonstrated that it significantly reduced the incidence of dysplasia (OR = 0.63), suggesting that inhibition of the JAK–STAT pathway can indirectly reduce chemokine overactivation [288] (Table 3).

Other inflammatory pathways may also represent potential therapeutic targets. The JNK inhibitor SP600125 suppresses abnormal intestinal epithelial proliferation by inhibiting the RIP3–JNK signaling axis and subsequently regulating CXCL1 expression, providing mechanistic support for pathway-directed intervention strategies [235].

#### Immune checkpoint-based combination strategies

The interaction between chemokine signaling and immune checkpoint pathways provides a rationale for combining chemokine-targeted therapies with immunotherapy. However, chemokine-mediated immune cell recruitment and exclusion mechanisms can influence therapeutic efficacy.

Immune checkpoint inhibitors (ICIs), particularly PD-1/PD-L1 blockade, have become established treatments for dMMR/MSI-H CRC [289, 290]. In the context of CAC, chronic inflammation may upregulate PD-L1 and create a potentially ICI-responsive milieu [291]. Moreover, chemokine signaling and immune checkpoint pathways are functionally intertwined: anti-PD-1 therapy can elevate tumor CXCL10 to recruit effector T cells, while CXCR3⁺ Tregs limit its efficacy. Therefore, combined targeting of CXCR3 and PD-1 inhibition may enhance therapeutic efficacy [283, 284] (Table 3).

Similarly, disrupting the CXCL12/CXCR4 axis with antagonists like AMD3100 can relieve immune exclusion and improve T-cell infiltration, thereby potentiating the response to anti-PD-1/PD-L1 therapies [251]. Thus, combining ICIs with chemokine-targeted strategies represents a rational and promising approach, though further CAC-specific studies are needed.

### Emerging regulatory strategies and therapeutic platforms

Unlike established molecularly targeted therapies, emerging strategies for CAC increasingly focus on remodeling the complex inflammatory microenvironment, regulating upstream disease-associated processes, or improving therapeutic delivery. Although many of these approaches have demonstrated promising efficacy in preclinical models, their clinical translation remains limited by challenges including delivery efficiency, specificity, safety, and long-term therapeutic benefit.

#### Epigenetic and RNA-based regulatory strategies

Chronic inflammation induces aberrant epigenetic alterations, including DNA methylation, histone modification, and dysregulated non-coding RNA expression, which contribute to CAC initiation and progression [11]. In CAC, several tumor suppressor genes, including p16 and members of the SFRP family, are frequently silenced through promoter hypermethylation, while inflammation-associated microRNAs participate in regulating tumor-promoting pathways.

For example, miR-21 is upregulated in both IBD and CAC, whereas miR-34b-5p is silenced through methylation. RNA-based therapeutic approaches, including miRNA mimics and antagomiRs, provide a potential strategy to restore abnormal regulatory networks [11]. However, despite their molecular specificity, RNA-based interventions remain in an exploratory stage because of challenges related to RNA stability, targeted delivery, immune activation, and clinical feasibility.

#### Microbiota-based and metabolic interventions

Microbiota-targeted therapy represents another promising preventive approach. Intestinal dysbiosis is closely associated with CAC development, and both enrichment of specific pathogenic bacteria and disruption of the overall microbial community can promote tumorigenesis through multiple mechanisms [292, 293].

Because broad-spectrum antibiotics may indiscriminately disrupt microbial balance, more selective strategies have been explored [285, 293]. For example, in the AOM/DSS model, oral sodium tungstate inhibited this pathway, reduced the abundance of specific Escherichia coli strains without markedly affecting obligate anaerobes, and was associated with reduced tumor incidence [285]. For chemokine upregulation driven by specific microbiota, intervention strategies include butyrate-rich dietary fibers and L. Rhamnosus GG, both of which exert effects through inhibition of the ETBF-STAT3–MCP-1 axis [294]. Additionally, oral mannose (2 g/kg) can modulate the TME by intervening in the lactate-MCP-1-TAM axis [295] (Table 3).

#### Nanotechnology-enabled therapeutic platforms

Nanotechnology-based platforms represent an emerging strategy that differs from conventional molecular targeting by focusing on improving the delivery, localization, and pharmacological properties of therapeutic agents [296]. Through enhanced permeability and retention effects and ligand-mediated targeting, nanoparticles can preferentially accumulate in inflamed or tumor tissues, thereby increasing local drug concentration while reducing systemic toxicity [297, 298].

Microenvironment-responsive nanoplatforms exploiting ROS, pH, or enzyme activity have also been developed to enhance local drug release and therapeutic efficacy. For example, the ROS-responsive B-ATK-T nanoprodrug showed synergistic antitumor effects in preclinical models [293, 299] (Table 3). Responsive nanoplatforms therefore represent a promising direction for improving drug delivery precision and therapeutic efficacy in CAC [300, 301]. Nevertheless, clinical translation remains challenging because of issues related to biodegradability, manufacturing complexity, and safety evaluation.

The preventive and therapeutic approaches discussed above highlight the potential of translating mechanistic insights into clinical strategies, while also underscoring the challenges that remain in achieving effective and durable control of CAC.

## Summary and perspectives

CAC is a multifactorial inflammation-driven malignancy arising from the interplay of gut microbiota dysbiosis, epithelial barrier disruption, persistent immune activation, oxidative stress, genetic and epigenetic alterations, and TME remodeling. These processes are interconnected rather than independent: microbial and barrier disturbances sustain inflammatory signaling, while chronic inflammation promotes DNA damage and molecular alterations that together facilitate tumor development and immune evasion. Within this network, chemokines and their receptors are important regulators of inflammatory amplification, immune-cell recruitment, and TME remodeling, but their effects are closely linked to the broader microbial, inflammatory, and immune context. This integrated view highlights the complexity of CAC pathogenesis and the need to consider multiple interconnected mechanisms when developing diagnostic and therapeutic strategies.

Several important gaps remain, particularly regarding the stage-specific and cell-specific functions of chemokine signaling. Chemokine activity may differ between chronic colitis, dysplasia, and established CAC, yet the temporal dynamics and cellular sources of individual chemokine axes remain insufficiently defined, especially in human tissues. Moreover, many proposed biomarkers and therapeutic strategies remain supported predominantly by preclinical evidence, limiting their clinical translation. Determining how individual chemokine axes function across different stages of CAC development, whether stage-specific chemokine signatures can improve risk stratification and disease surveillance, and whether selective modulation of these axes can prevent tumor development or improve treatment of established CAC without compromising protective immunity therefore remains an important challenge for the field.

Addressing these questions will require longitudinal clinical cohorts, rigorous biomarker validation, and integration of single-cell and spatial transcriptomic approaches to resolve stage and cell-specific chemokine networks. Such studies may help distinguish biomarkers with diagnostic or prognostic value from those that are therapeutically actionable and clarify when chemokine modulation is most appropriate for prevention or treatment. Taken together, current evidence supports chemokine signaling as an important component of the broader inflammatory network underlying CAC, while its clinical relevance depends on defining stage-specific functions and validating their utility in human populations.

## Data Availability

Not applicable.

## Abbreviations

IBD: Inflammatory bowel disease
UC: Ulcerative colitis
CD: Crohn's disease
CRC: Colorectal cancer
CAC: Colitis-associated colorectal cancer
TME: Tumor microenvironment
GPCRs: G protein-coupled receptors
MDSCs: Myeloid-derived suppressor cells
Tregs: Regulatory T cells
ELR: Glutamate-leucine-arginine
PLC: Phospholipase C
PI3K: Phosphatidylinositol 3-kinase
MAPK: Mitogen-activated protein kinase
NF-κB: Nuclear factor kappa-light-chain-enhancer of activated B cells
EMT: Epithelial-mesenchymal transition
CAFs: Cancer-associated fibroblasts
TAMs: Tumor-associated macrophages
KRT17: Keratin 17
ROS: Reactive oxygen species
MCP-1: Monocyte chemoattractant protein-1
PP1: Protein phosphatase 1
PP2A: Protein phosphatase 2A
Akt: Protein kinase B
mTOR: Mammalian target of rapamycin
PDK1: Phosphoinositide-dependent kinase 1
STAT3: Signal transducer and activator of transcription 3
JAK: Janus kinase
CKIs: CDK inhibitors
CDKs: Cyclin-dependent kinases
APC: Adenomatous polyposis coli
TCF: T-cell factor
LEF1: Lymphoid enhancer-binding factor 1
SDF-1: Stromal cell-derived factor-1
TCR: T-cell receptor
DSS: Dextran sulfate sodium
5-ASA: 5-Aminosalicylic acid
DHA: Docosahexaenoic acid
SARI: Suppressor of AP-1 and regulated by IFN
siRNA: Small interfering RNA
AAV8: Adeno-associated virus serotype 8
PLGA: Poly(lactic-co-glycolic acid)
ETBF: Enterotoxigenic Bacteroides fragilis
LGG: Lactobacillus rhamnosus GG
HK-II: Hexokinase II
COX-2: Cyclooxygenase-2
Fn: Fusobacterium nucleatum
YTHDF2: YTH N6-methyladenosine RNA binding protein 2
JNK: c-Jun N-terminal kinase
7ND: N-terminal deletion mutant of CCL2
